# Design, Synthesis, and Biological Evaluation of N-Acyl-Homoserine Lactone Analogs of Quorum Sensing in *Pseudomonas aeruginosa*

**DOI:** 10.3389/fchem.2022.948687

**Published:** 2022-07-08

**Authors:** Zhenyu Wei, Ting Li, Yan Gu, Qian Zhang, Enhui Wang, Wenbo Li, Xin Wang, Yang Li, Hongyu Li

**Affiliations:** ^1^ International Scientific and Technological Cooperation Base of Biopharmaceutical, School of Life Sciences, Institute of Microbiology, Lanzhou University, Lanzhou, China; ^2^ Gansu High Throughput Screening and Creation Center for Health Products, School of Pharmacy, Lanzhou University, Lanzhou, China

**Keywords:** AHL analogs, quorum sensing, *Pseudomonas aeruginosa*, anti-virulence, antibiofilm

## Abstract

Quorum sensing plays a necessary role in the production of virulence factors and the formation of biofilm on *Pseudomonas aeruginosa*. Thus, the development of inhibition of quorum sensing is one of the most promising methods to control bacterial infection and antibiotic resistance. In this work, nine novel AHL analogs were designed, synthesized, and evaluated as potential quorum sensing inhibitors. The results depicted that structural modifications have significant effects on quorum sensing inhibition activity of AHL molecules. Without inhibiting the growth of *P. aeruginosa*, 2-(4-bromophenyl)-N-(2-oxotetrapyridinefuran-3-yl) butanamide (compound no.10) showed the excellent performance in inhibiting biofilm formation and virulence factor production among all the compounds through robustly suppressing the expression of QS related genes. In a molecular docking study, compound no.10 exhibited a higher affinity toward LasR than other AHL analogs. In addition, compound no.10 also exhibits the best inhibition effect on virulence production in the *Caenorhabditis elegans* infection model.

## 1 Introduction

*Pseudomonas aeruginosa* as an important nosocomial pathogen could threaten those who are long-term intubated and immunocompromised, and patients suffering from cystic fibrosis, traumatized cornea, burns, Gustilo open fractures, and lead to lethal infections (Hogardt and Heesemann, 2013; Moradali et al., 2017). A previous study reported that biofilm formation and virulence factor production are the major causes of *P. aeruginosa* infections (Schuster and Greenberg, 2006). At present, antibiotics such as tobramycin, meropenem, or ciprofloxacin, are considered the first choice in treating infections of *P. aeruginosa*. However, the wild use of antibiotics to treat infections of *P. aeruginosa* has led to the generation of multidrug-resistant pathogens (Abbas et al., 2017). Thus, combating *P. aeruginosa* infections need a new target.

Quorum sensing (QS) is a bacteria-to-bacteria communication system. Also, QS is moderated by the production of different kinds of signal molecules known as autoinducers, which are usually N-acyl-homoserine lactones (AHLs) in Gram-negative bacteria (Ng and Bassler, 2009). In *P. aeruginosa*, the QS system is composed of four interconnected systems (Las, Rhl, PQS, and IQS), among them, the LasI/LasR system is the most representative and primary (Hançer Aydemir et al., 2018). In addition, each system utilizes a particular signal molecule such as N-(3-oxododecanoyl)-L-homoserine lactone (OdDHL), N-butanoyl-L-homoserine lactone (BHL), 2-heptyl-3-hydroxy-4-quinolone, and 2-(2-hydroxyphenyl)-thiazole-4-carbaldehyde (Hao et al., 2021). Typically, the accumulation of signal molecules interacts with a transcriptional activator protein (Abbas et al., 2017), and then the expression of QS-regulated genes were activated (Schuster and Greenberg, 2006).

It is reported that pathogenicity of *P. aeruginosa* is generally related to virulence production and biofilm formation (Bassler and Losick, 2006; Hançer Aydemir et al., 2018). As QS inhibitors (QSIs) had no influence on bacterial growth, they are believed to generate weaker selection for antibiotic resistance than conventional antibiotics. Thus, QSIs can be a novel and promising way to attenuate *P. aeruginosa* infection (Hançer Aydemir et al., 2018). Nowadays, numerous QS signal molecule analogs and plant-derived natural substances have been reported as QSIs to interfere with the QS system of *P. aeruginosa* (Qu et al., 2016; Abbas et al., 2020; Hao et al., 2021; Saqr et al., 2021). Unfortunately, its dose-dependent toxicity and a weak effect significantly limit clinical applications. Therefore, development of novel safe and effective QSIs is urgently needed for the treatment of *P. aeruginosa* infections.

In this study, nine novel AHL analogs were designed, synthesized, and evaluated as potential QSIs against *P. aeruginosa*. The effects of AHL analogs on biofilm formation and virulence factor production *in vitro* were evaluated. Furthermore, the mechanism was studied by related gene expression and molecular docking.

## 2 Materials and Methods

### 2.1 Strains and Media

*Pseudomonas aeruginosa* PAO1 and *Escherichia coli* OP50 were stored in our laboratory. *Cae*norhabditis *elegans* N2 were obtained from the Caenorhabditis Genetics Center (CGC). *P. aeruginosa* PAO1 was cultured using Luria–Bertani broth (LB) at 37 °C and maintained on a nutrient agar plate (Solarbio, Beijing, China) at 4 °C. *C*. *elegans* was cultured using the nematode growth medium (NGM) at 20 °C and *E. coli* OP50 was used as the standard food source. 4-Br-PHL (compound no.1) and other AHL analogs (compound no.2 to compound no.10) were dissolved in DMSO before treatment (Solarbio, Beijing, China).

### 2.2 Synthesis of Acyl-Homoserine Lactone (AHL) Analogs

**FIGURE 1:**
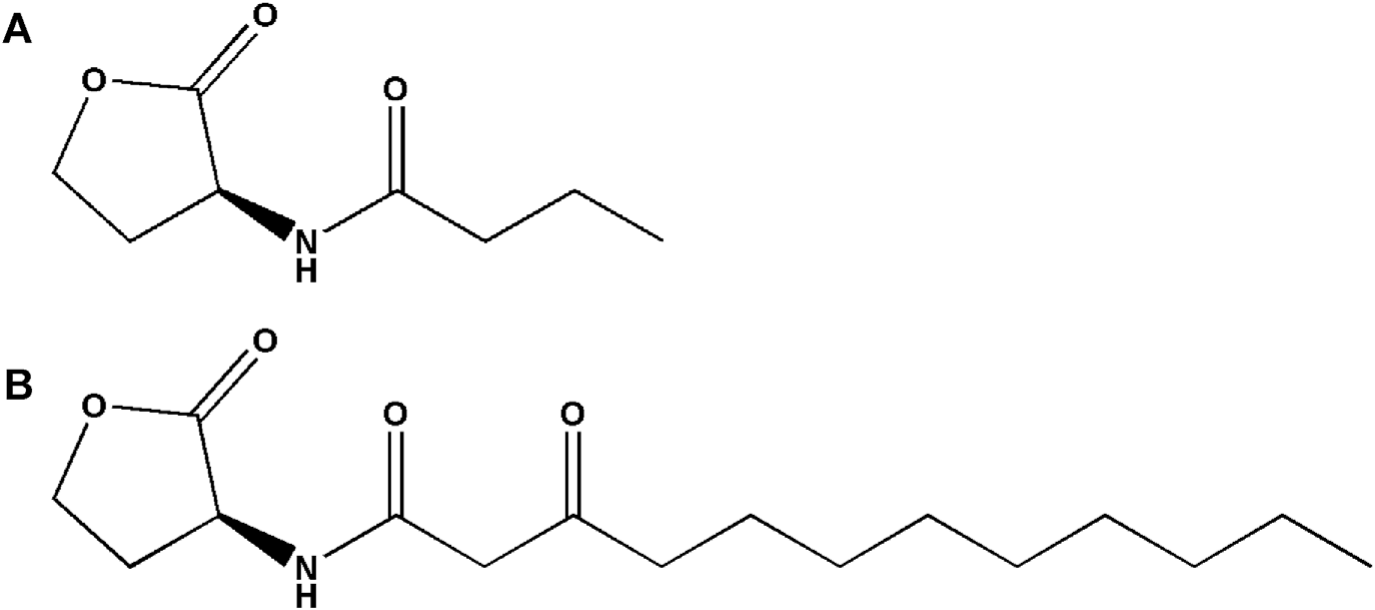
Chemical structure of P. aeruginosa PAO1 natural signal molecule. **(A)** Structure of BHL and **(B)** structure of OdDHL.

**FIGURE 2:**
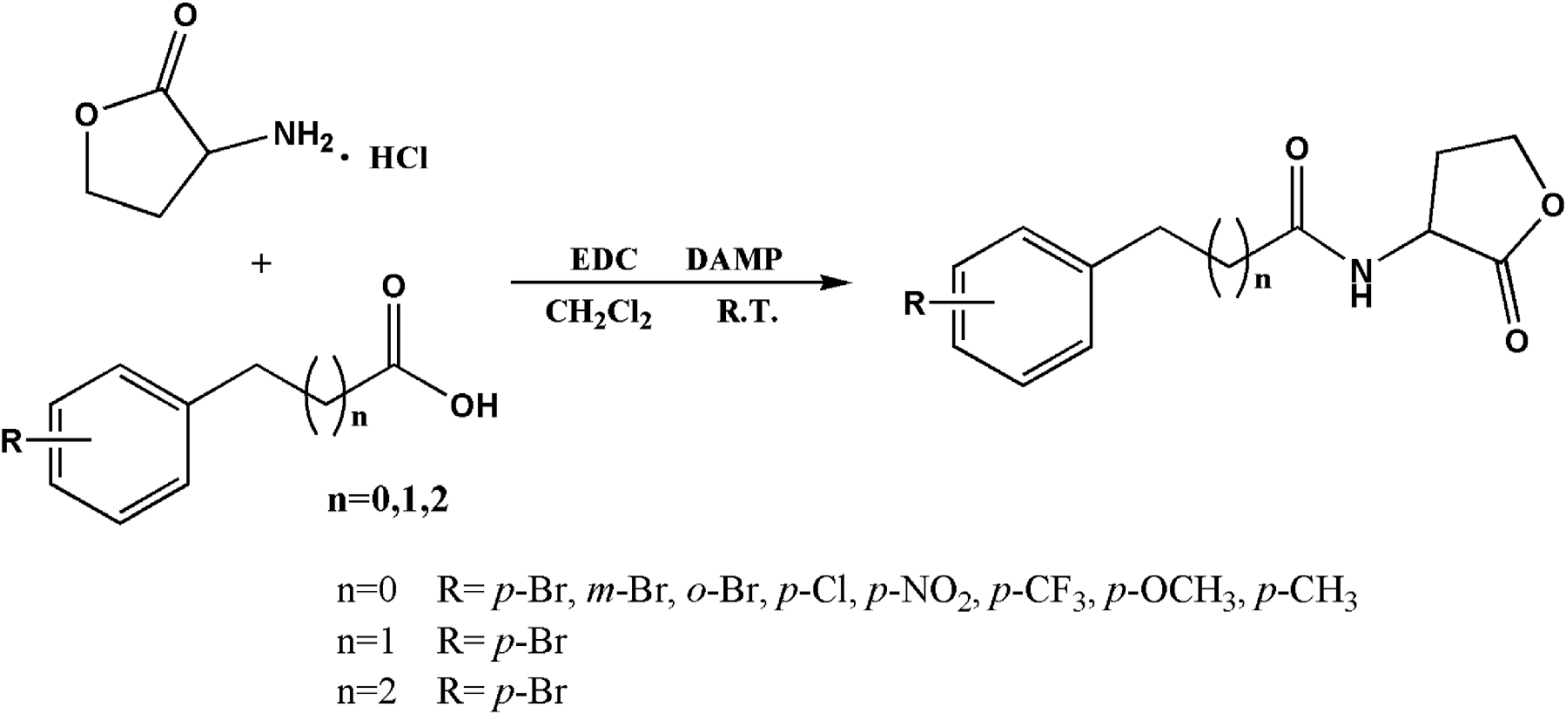
Synthesis of AHL analogs. RT = room temperature.

Based on the structures of OdDHL and BHL (Figure 1), we synthesized a series of novel AHL-based QSIs, in which the natural homoserine lactone ring is present but the natural acyl group was substituted. The structures of analogs were modified from three aspects: 1) the acyl group, 2) the length of the acyl side chain, and 3) the type and position of the substituents on the benzene ring. The synthesis method is shown in Figure 2. Those AHL analogs used in this study were synthesized by us and characterized by NMR, and MS with a purity of ≥95% (Supplementary Table S1). To circumvent the issues of different assessment results, we also synthesized N-(4-bromophenylacetanoyl)-L-homoserine lactone (4-Br-PHL, no. 1) as a positive control (Geske et al., 2005).

### 2.3 Initial Screening of Analogs

To initial screen compounds with QS-inhibited activity, QS-regulated biofilm formation was tested as the screening indicator. As described previously (Luo et al., 2017), biofilm mass was measured using a crystal violet assay.

### 2.4 Planktonic Cell Growth

The planktonic cell growth assay was slightly modified (Qu et al., 2016). Overnight cultures of *P. aeruginosa* PAO1 were diluted in LB broth. The suspension was supplemented with two compounds (no.3 and no.10) with concentrations ranging from 10 to 400 μM and incubated at 37 °C. Then, 1 ml samples were obtained every 2 h, and the turbidity was recorded at 600 nm using a spectrophotometer (Thermo scientific, Finland), then a growth curve was generated.

### 2.5 Biofilm Inhibition Analyses

In this assay, the inhibitory effects of compound no.3 and compound no.10 on biofilm formation were further identified at different concentrations ranging from 50 to 400 μM. The method of this assay is similar to the method of initial screening of analogs. In addition, cover slides were incubated statically with or without 200 μM compound no.3 and compound no.10 at 37 °C to facilitate cell attachment and biofilm formation. Also, compound no.1 was conducted as a positive control. Finally, the structures of biofilm were observed using an upright microscope (BX53, Olympus, Japan) at ×20 magnification.

### 2.6 Motility Assay

#### 2.6.1 Swimming

The swimming assay method was modified from Rashid and Kornberg (2000). Swimming agar plates (10 g/L peptone, 5 g/L NaCl, and 0.3% agarose) with compound no.3 and no.10 (200 μM) and blank control plates were center stabbed with *P. aeruginosa* PAO1 using sterile toothpick. Also, compound no.1 was conducted as a positive control. After incubating at 30 °C for 12–14 h, swimming zones were determined by measuring the diameter of circular expansion.

#### 2.6.2 Swarming

The swarming plate was composed of nutrient agar (8 g/L) and glucose (5.0 g/L). Then, swarming plates with and without supplementation of 200 μM compound no.3 and no.10 were added 5 μl overnight culture of *P. aeruginosa* PAO1. In addition, compound no.1 was conducted as a positive control. Then, the swarming plates were placed at 30 °C and incubated for 18–24 h (Kumar et al., 2013), and swarming zones were determined by measuring the diameter of circular expansion.

#### 2.6.3 Twitching

Twitching motility of *P. aeruginosa* PAO1 was modified from Saqr et al. (2021). A measure of 1% of LB agar with or without compounds (compound no.3 and compound no.10, 200 μM) stabbed with a toothpick up to bottom of the Petri dish from overnight culture *P. aeruginosa* PAO1 in order to evaluate twitching motility inhibition. Compound no.1 was conducted as a positive control, the agar was removed after incubation at 37 °C for 48 h, and then the plates were dried in air and stained with crystal violet. The dye was washed off with sterile water and the twitching zones were measured.

### 2.7 Pyocyanin Analyses

The assay of the inhibitory effect of compound no.3 and compound no.10 on pyocyanin production was conducted using previous methods (O'Loughlin et al., 2013). Overnight, *P. aeruginosa* PAO1 cultures were subcultured into LB broth with the compound no.3 and compound no.10 at the concentration of 200 μM for endpoint assays following 24 h of aerobic growth with shaking at 37 °C. Also, compound no.1 was conducted as a positive control. Finally, the production of pyocyanin was measured by recording the absorbance of this pink layer at 520 nm.

### 2.8 Elastase Analysis

The skim milk agar method (Hao et al., 2021) was used to determine the effect of compound no.3 and compound no.10 on elastase activity. Also, compound no.1 was conducted as a positive control. After overnight culturing, *P. aeruginosa* PAO1 with or without compound no.3 and compound no.10 were incubated at 37 °C for 24 h at 180 rpm. After 24 h , the cultures were centrifuged at 10,000 rpm for 15 min. A measure of 100 μl of supernatant was added to the wells of skim milk agar and then plates were incubated for 24 h at 37 °C. The elastase activity was determined by measuring the diameter of clear zone.

### 2.9 *C. elegans* Life Span Assays

To further investigate the anti-virulence effect of compound no.3 and compound no.10, we used a *C. elegans* life span assay. Also, compound no.1 was conducted as a positive control. This assay was modified from a previous study (Cezairliyan et al., 2013; O'Loughlin et al., 2013). After synchronization, *C. elegans* was grown to the L4 stage at 20 °C. In addition, an equivalent volume of compound no.1, compound no.3, and compound no.10 were mixed to the *P. aeruginosa* PAO1 cultures during growth and DMSO was added as a blank control. Then, an overnight culture of *P. aeruginosa* PAO1 was poured on Petri plates, containing PGS agar (1% peptone, 1% NaCl, 1% glucose, 150 mM sorbitol, and 1.7% agar) and kept for 24 h incubation at 37 °C. After 8–24 h, 25–30 L4 stage *C. elegans* were seeded with each plate. Then, live or dead worms were scored every 3 h at 25 °C and again at 24 h.

### 2.10 Gene Expression Analysis

According to the previous study (Qu et al., 2016), the expression of key QS regulatory genes (*lasI*, *lasR*, *rhlI*, *rhlR*, and *mvfR*) of *P. aeruginosa* PAO1 was monitored in the presence of compound no.3 and compound no.10. Also, compound no.1 was conducted as a positive control. The primers for the genes are shown in Supplementary Table S2. The 16S rRNA housekeeping gene was served as an internal control. Total RNA was isolated with an Ultrapure RNA kit (CWBIO, Beijing, China), and the first-strand cDNA was synthesized with a Hifair^®^ Ⅲ 1st Strand cDNA Synthesis SuperMix for qPCR (Yeasen, Beijing, China) according to the manufacturer’s instructions. The reaction was carried out with Hieff UNICON^®^ Universal Blue qPCR SYBR Green Master Mix (Yeasen, Beijing, China) also according to the manufacturer. The relative gene expression was calculated using the 2^−ΔΔCt^ method.

### 2.11 Molecular Docking Study

Molecular docking was conducted to analyze the interaction of compound no.3 and compound no.10 with the LasR receptor of *P. aeruginosa* PAO1. The crystal structure of *P. aeruginosa* PAO1 LasR was obtained from the Protein Data Bank (PDB ID:2UV0) (Bottomley et al., 2007). Hydrogen atoms were added according to the hydrogen network optimization and then minimized with an Amber19SB force field with UCSF Chimera. Finally, the protein structure was converted to a pdbqt file by prepare_receptor4.py script using AutoDockTools, and all the hydrogen atoms were kept. The ligands structures were built with UCSF Chimera, minimized with the GAFF force field, and converted to a pdbqt format by using the prepare_ligand4.py script with all hydrogen atoms. The docking site was determined by the *P. aeruginosa* PAO1 binding pocket. Finally, the molecular docking was performed by watvina (https://www.github.com/biocheming/watvina), which was optimized with new scorning function, with van der Waals, hydrogen bond, polar repulsion, and weak hydrogen bonds contribution. Conformation searching was performed by a simplified genetic algorithm and simulated annealing mixed with BFGS local optimization. Finally, the docking pose was optimized while the protein was kept fixed.

### 2.12 Statistical Analysis

All experiments were conducted in triplicate to validate the reproducibility. All values are presented as the mean ± standard error. One-way ANOVA was performed using SPSS 23.0 software. The significance was accepted when the *p*-value was less than 0.05. Graphs were constructed using GraphPad Prism 7.0 software.

## 3 Results

### 3.1 AHL and Its Analogs

A series of novel AHL-based QS inhibitors are presented in Table 1. All compounds were characterized by NMR, MS with a purity of ≥95% (Supplementary Table S1).

**TABLE 1:** Inhibitory rate of AHL analogs.

Compound	Structure	Concentration (μM)	Inhibitory rate
No.1	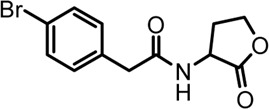	100	6.22 ± 2.77%
No.2	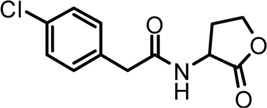	100	17.04 ± 2.56%
No.3	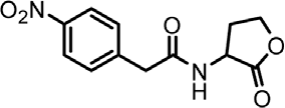	100	36.78 ± 0.18%
No.4	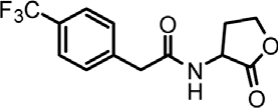	100	10.54 ± 3.51%
No.5	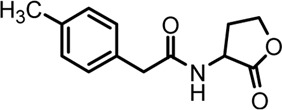	100	5.11 ± 1.65%
No.6	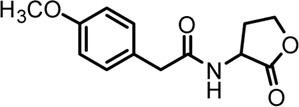	100	−20.09 ± 0.29%
No.7	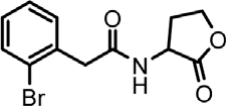	100	7.78 ± 1.46%
No.8	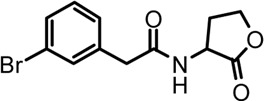	100	5.47 ± 1.67%
No.9	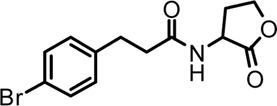	100	17.46 ± 1.25%
No.10	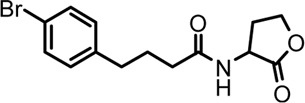	100	37.46 ± 2.31%

### 3.2 Preliminary Screening of Analogs

The biofilm formation inhibition activity of compounds 1–10 was assessed. As shown in Table 1, all compounds showed inhibitory activity against biofilm formation of *P. aeruginosa* PAO1, except for compound no.6. Among all analogs, compound no.3 and compound no.10 exhibited the best inhibition activity and were identified as the potential QS-inhibitor to further evaluate in the following experiments.

### 3.3 Planktonic Cell Growth

**FIGURE 3:**
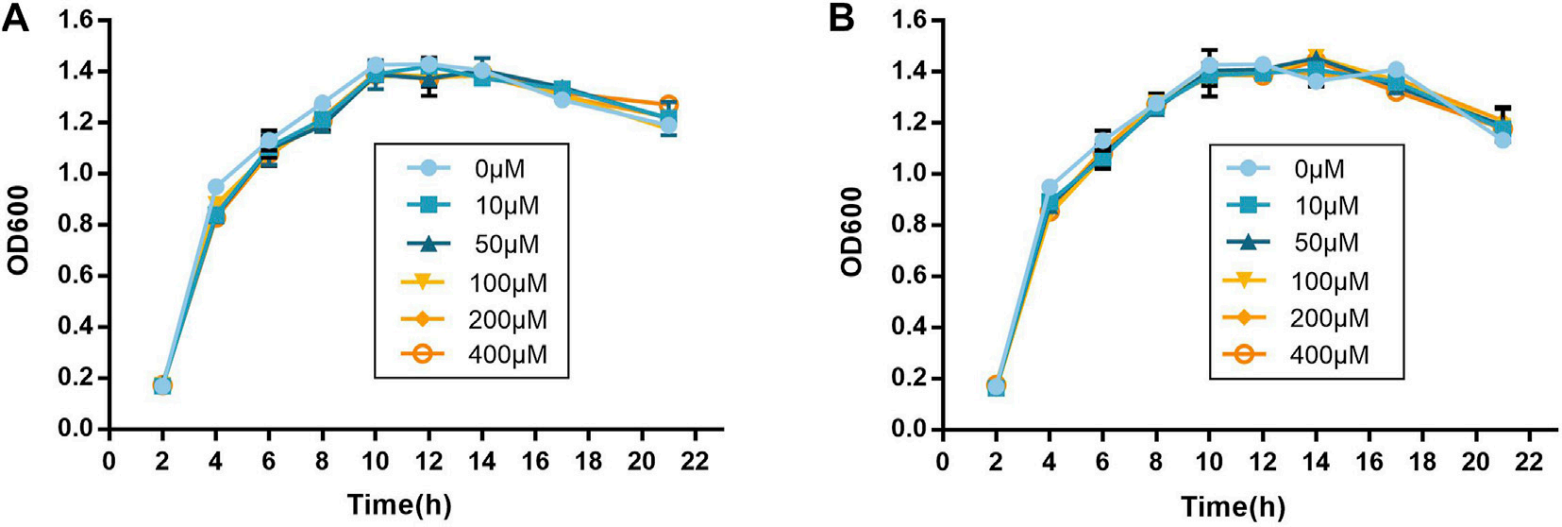
Inhibitory effect of compound no.3 and compound no.10 on **(A)** pyocyanin and **(B)** elastase productions of P. aeruginosa PAO1.

The growth curves were drawn to identify the influence on the growth of *P. aeruginosa* PAO1. As shown in Figure 3, compound no.3 and compound no.10 had no inhibition effect on growth of *P. aeruginosa* PAO1 at the concentrations ranging from 10 to 400 μM.

### 3.4 Biofilm Inhibition Analyses

**FIGURE 4:**
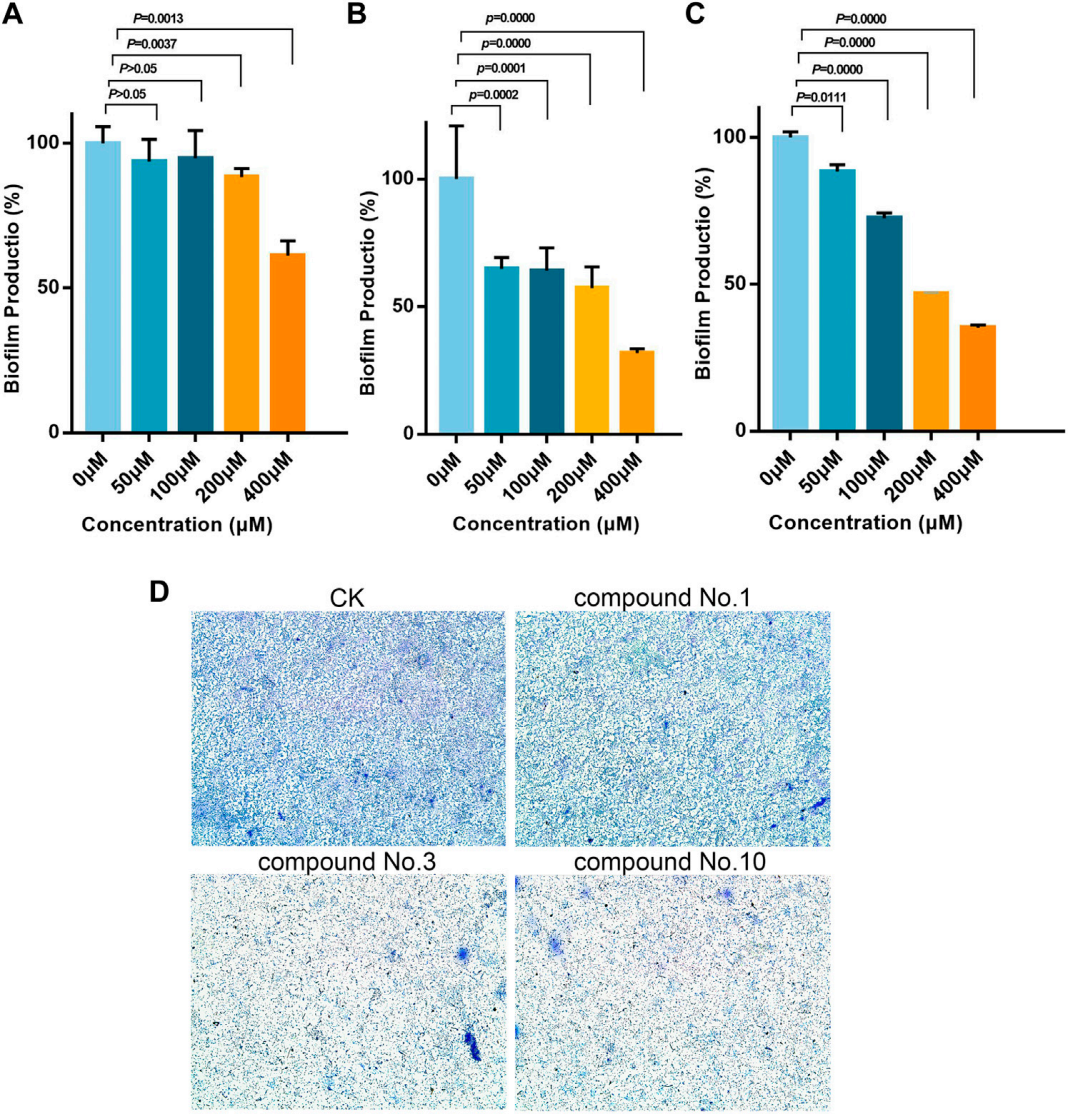
Inhibitory effect of compound no.3 and compound no.10 on *P. aeruginosa* biofilm formation. Biofilm was stained by crystal violet and measured as absorbance at 570 nm. **(A)** Compound no.1, **(B)** compound no.3, **(C)** and compound no.10. **(D)** Representative microscope images of treated with compounds at 200 μM.

As shown in Figures 4A–C, inhibitory activities of compound no.3 and compound no.10 on biofilm formation were dose-dependent. Compound no.1 had slightly reduced biofilm formation at the concentration of 200 μM. By contrast, compound no.3 and compound no.10 had more obvious inhibitory effect on biofilm formation at the concentrations ranging from 200 to 400 μM. In the presence of compound no.3, the biofilm formation of *P. aeruginosa* PAO1 was decreased to nearly 35% at the concentration ranging from 50 to 400 μM. Remarkedly, compound no.10 inhibited more than 60% of biofilm formation at above 200 μM.

The aforementioned results were further revealed under a microscope. From Figure 4D, reduction of biofilm attached to the surface of plates was observed in treated groups. Compared with compound no.1, the reduction of biofilms was more obvious in compound no.3 and compound no.10 treated groups.

### 3.5 Motility Assay

**FIGURE 5:**
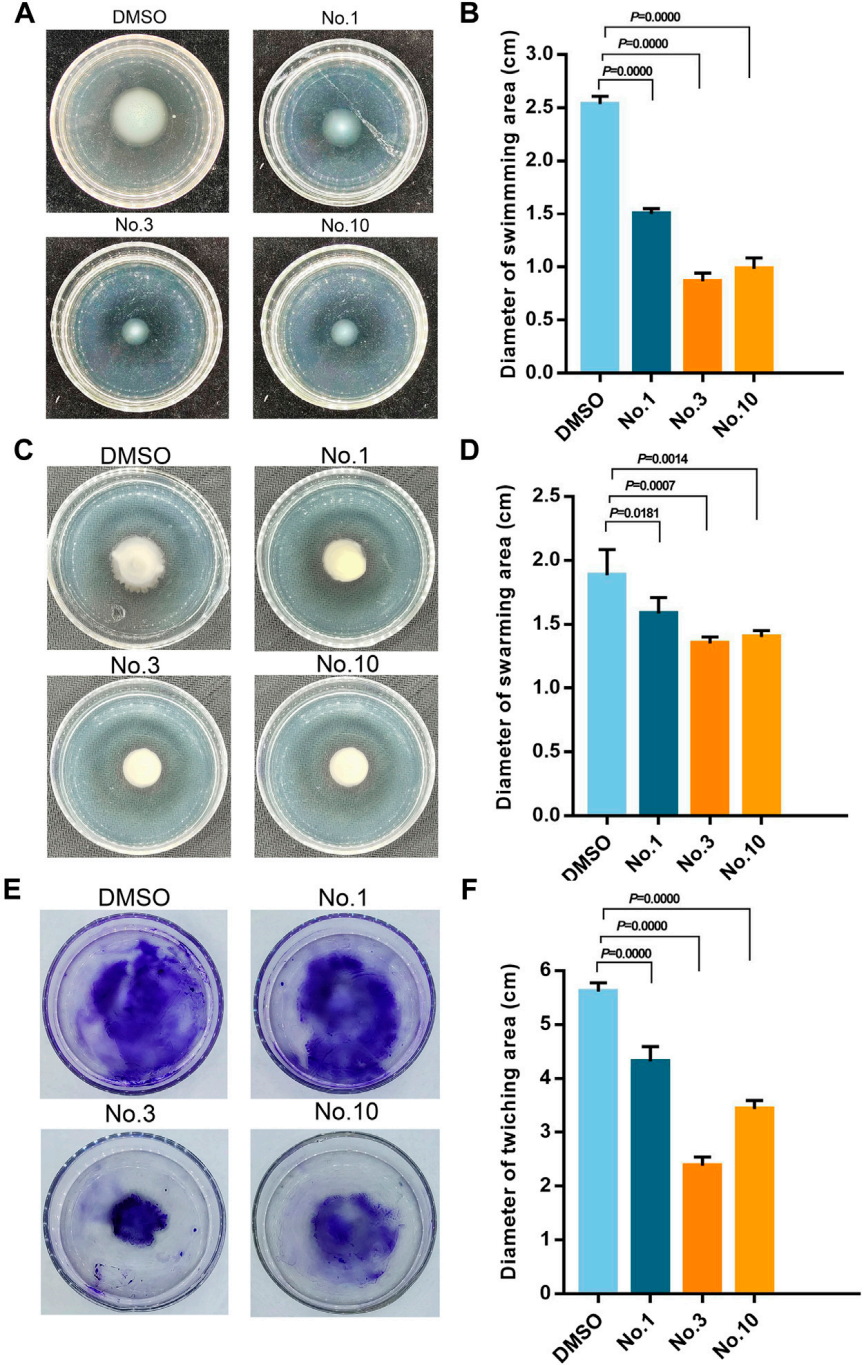
Inhibitory effect of compound no.3 and compound no.10 on *P. aeruginosa* motility. **(A)** and **(B)** represent swimming motility of *P. aeruginosa* PAO1, **(C)** and **(D)** represent swarming motility of *P. aeruginosa* PAO1, **(E)** and **(F)** represent twitching motility of *P. aeruginosa* PAO1. DMSO and compound no.1 represent negative and positive control, respectively.

As shown in Figure 5, in the absence of compounds, the swimming, swarming, and twitching zones for *P. aeruginosa* PAO1 are 2.53 ± 0.08 cm, 1.88 ± 0.20 cm, and 5.62 ± 0.16 cm, respectively. Compound no.1 exerted a weak inhibitory effect that the swimming, swarming, and twitching zones are 1.5 ± 0.05 cm, 1.58 ± 0.13 cm, and 4.32 ± 0.28 cm. In the presence of compound no.3, it resulted in a significant decrease in motility zones to 0.87 ± 0.07 cm, 1.35 ± 0.13 cm, and 2.38 ± 0.16 cm. Moreover, compound no.10 also reduce the motility zones to 0.98 ± 0.10 cm, 1.40 ± 0.05 cm, and 3.43 ± 0.16 cm, respectively.

### 3.6 Pyocyanin Analyses

**FIGURE 6:**
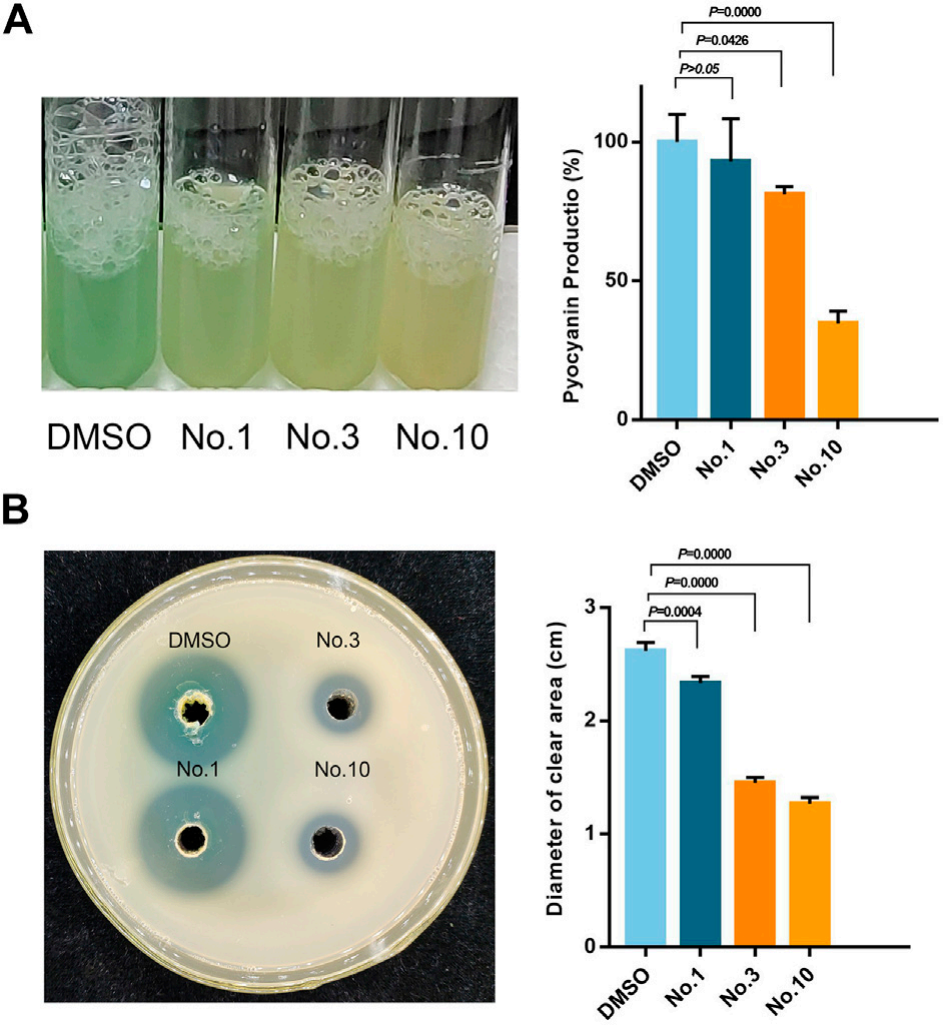
Inhibitory effect of compound no.3 and compound no.10 on **(A)** pyocyanin and **(B)** elastase productions of *P. aeruginosa* PAO1.

Pyocyanin is a green exotoxin generated by *P. aeruginosa* governed by the QS system. The effect of compounds on pyocyanin production was studied using the chloroform extraction assay. As shown in Figure 6A, compound no.1 and compound no.3 exhibited a weak inhibitory effect. By contrast, compound no.10 robustly lowered pyocyanin production by 65.29%.

### 3.7 Elastase Analysis

The effect of compounds on elastase activity was assessed with the skim milk agar method. As shown in Figure 6B, all the compounds can effectively inhibit the activity of elastase compared with the control group. Compared with compound no.1, significant reduction in the diameter of the clear zone was produced in compound no.3 and no.10 treated groups on a skim milk plate.

### 3.8 Effect on the *C. elegans* Life Span

**FIGURE 7:**
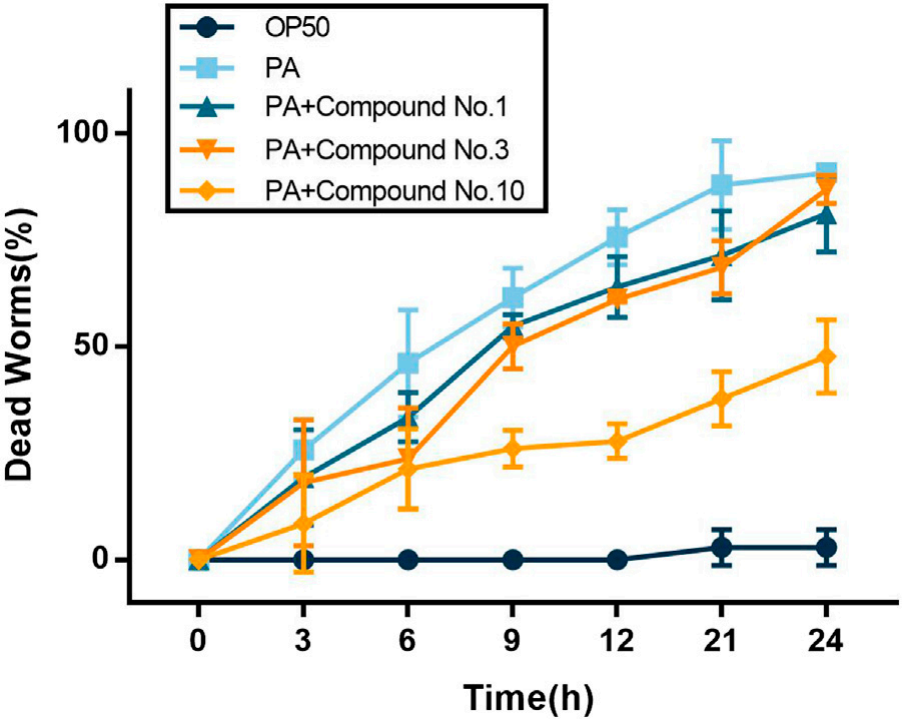
Compound no.3 and compound no.10 decreased toxicity of *P. aeruginosa* PAO1 in the *C. elegans* model.

As shown in Figure 7, at 0–6 h, compound no.3 and compound no.10 exhibited an excellent effect on the survival rate compared with compound no.1. Then, the effect of compound no.10 was more obvious in improving the survival rate of infected *C. elegans* than no.3 at the 6–24 h. Compared with the untreated group, the survival rate of *C. elegans* of the compound no.10 group reaches nearly 80% within 6 h of incubation and can still be reached to 52% within 24 h.

### 3.9 Effect on Expression of QS-Regulated Genes

**FIGURE 8:**
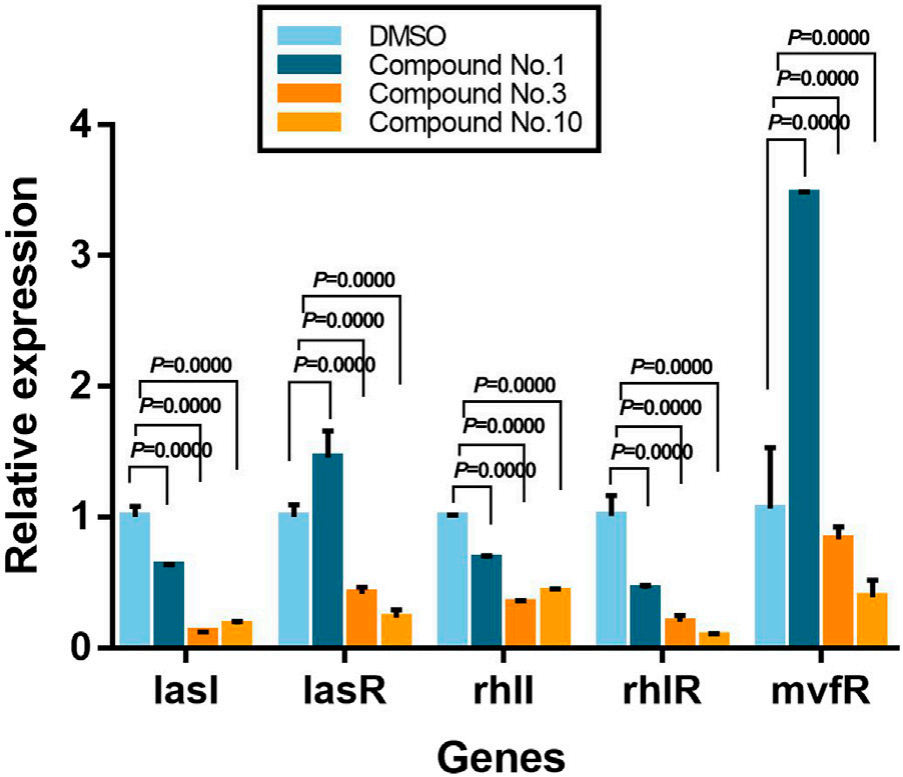
Relative expression levels of QS-regulated genes in the presence of compound no.1, compound no.3, and compound no.10 as determined by real-time quantitative PCR.

To further determine the inhibition mechanism of compounds on *P. aeruginosa* PAO1 virulence and biofilm formation, the expression level of key QS-related genes was assessed using qPCR. From Figure 8, it shows that compound no.3 and no.10 downregulated the expression of *lasI*, *lasR*, *rhlI*, *rhlR*, and *mvfR*. Compared with the untreated group, compound no.1 slightly inhibited the expression of *lasI*, *rhlI*, and *rhlR*. The relative expression of QS regulatory gene treatment with compound no.3 decreased by 87% for *lasI*, 59% for *lasR*, 66% for *rhlI*, 80% for *rhlR*, and 17% for *mvfR*, respectively, and compound no.10 decreased 82% for *lasI*, 77% for *lasR*, 57% for *rhlI*, 91% for *rhlR*, and 61% for *mvfR*, respectively.

### 3.10 Molecular Docking

**FIGURE 9:**
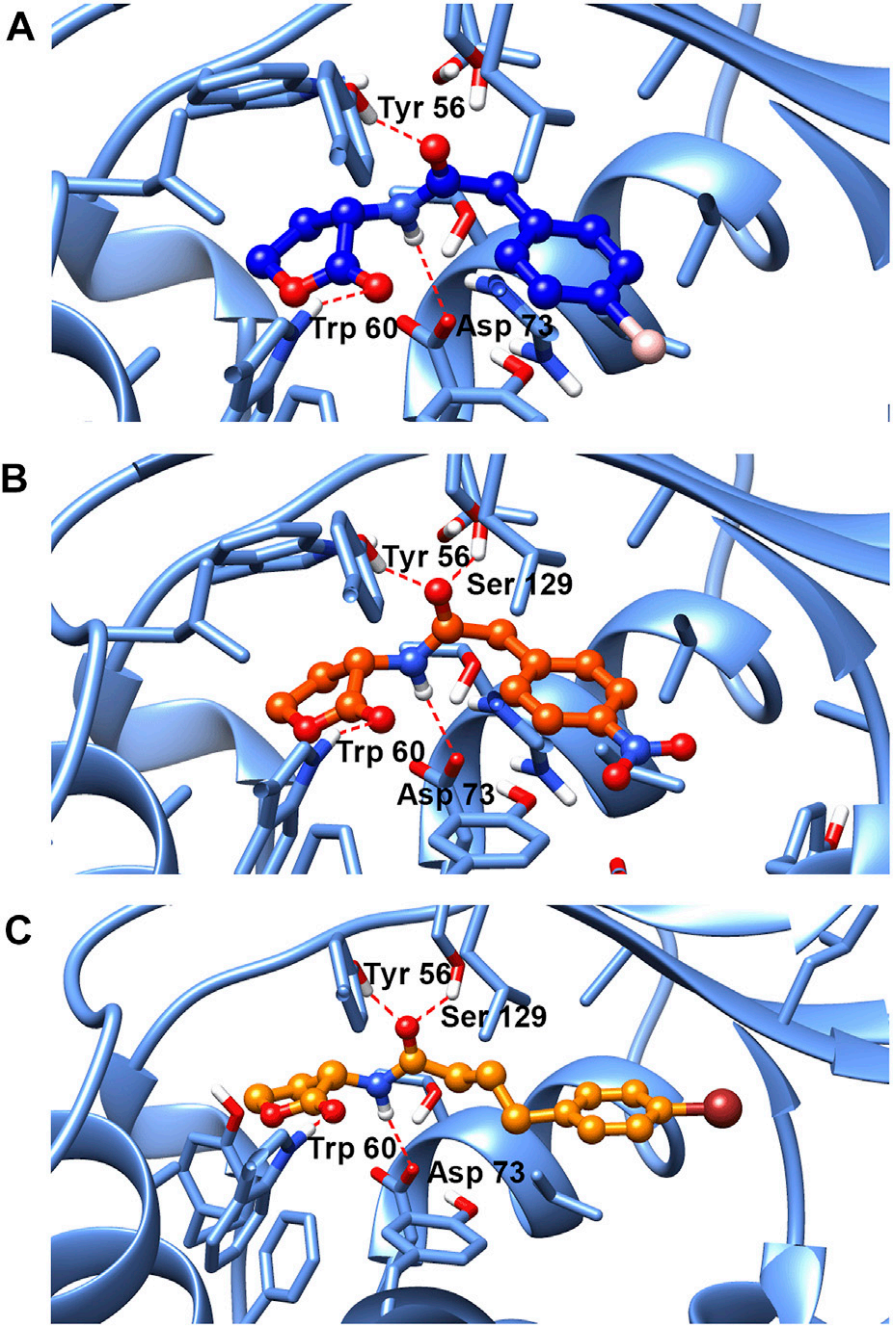
Molecular docking of **(A)** compound no.1, **(B)** compound no.3, and **(C)** compound no.10 into the active site of LasR. Both compound no.3 and compound no.10 interacted with LasR by formation of H-bonding with the Try 56, Trp 60, Asp 73, and Ser 129. Compound no.1 interacted with LasR with Try 56, Trp 60, and Asp 73 by H-bonding.

To further explore the binding mode of compounds to the LasR, molecular docking analysis of compounds no.1, no.3, and no.10 was performed. As shown in Figure 9, the interaction between compound no.1 and LasR by formation of H-bonding with Try 56, Trp 60, and Asp 73. It may be the reason that compound no.1 had weaker effects on biofilm formation, motility, and virulence production of *P. aeruginosa* PAO1. By contrast, the interaction between compounds (no.3 and no.10) and LasR receptor by formation of H-bonding with the Try 56, Trp 60, Asp 73, and Ser 129, is consistent with the binding sites of OdDHL (Abbas et al., 2017). The Lavina scores indicated a higher affinity of compound no.10 to bind with LasR than compound no.3 resulting in a more considerable inhibition activity of biofilm formation and virulence production.

## 4 Discussion

It is known that the pathogenicity of *P. aeruginosa* is contributed to the virulence factor production and the biofilm formation, which could lead to lethal infection and antibiotics defense (Costerton, 2001; Clatworthy et al., 2007; Zhao and Liu, 2010). Research evidence indicated that QS of *P. aeruginosa* was closely related to these phenomena (Lee and Zhang, 2015; Hegazy et al., 2021). Indeed, the structures of natural autoinducers have often served as an intermediate for the synthesis of the QS inhibitor (Galloway et al., 2011; O'Loughlin et al., 2013). In this study, we designed and synthesized nine novel AHL analogs and evaluated their inhibition activities and revealed their mechanism in the QS system of *P. aeruginosa*.

As described previously, subtle alterations to substituents and their placement on the AHL acyl group dramatically influenced analog inhibitory activity (Hodgkinson et al., 2012). Also then, the inhibitory activity of AHL analogs is highly dependent on the structure and position of substituents in the benzene group (Geske et al., 2008; Abbas et al., 2017). In this study, the chemical modification of AHL analogs is initiated by the replacement of the hydrocarbon chain length and by replacing substituents on the benzene ring, then nine novel AHL analogs were designed and synthesized.

In the initial screening assay, by using the QS-regulated production of biofilm as the screening indicator, compound no.3 and compound no.10 were selected to conduct further investigation. Consistent with the biofilm inhibition results, compound no.3 and compound no.10 also significantly inhibited the swimming, swarming, and twitching ability compared with compound no.1 at the concentration of 200 μM. In a previous study, the biofilm formation and adhesion to tissue related to the motility of bacteria were confirmed (Landry et al., 2006). Furthermore, it is reported that thin and dispersed biofilm was formed by QS deficient *P. aeruginosa* (Lee et al., 2018; Mayer et al., 2020). Therefore, we supposed that the inhibition in motility is one of the reasons for biofilm formation inhibition by compound no.3 and compound no.10.

*P. aeruginosa* is armed by an armory rich in virulence factors that are regulated by QS and pyocyanin and elastase are representative virulence of *P. aeruginosa* (Chatterjee et al., 2016). After being treated with compound no.3 and compound no.10, the production of pyocyanin was reduced by nearly 19 and 66%, respectively. The activity of elastase was also inhibited by 44 and 52%. Compound no.10 had a more obvious effect on inhibiting the virulence production of PAO1 *in vitro*. To further illustrate the inhibitory effect of those compound no.1, compound no.3, and compound no.10 on virulence production, *C. elegans* life span assays were conducted (Utari and Quax, 2013). Compared with compound no.3, compound no.10 had a more continuous and significant effect on improving the survival rate of infected *C. elegans*.

Moreover, compound no.10 exhibited more inhibition activity on pyocyanin production compared with other AHL analogs with a non-native tail region and native head group reported by previous studies (Morkunas et al., 2012). Chlorolactone (CL) was synthesized by Swem et al. (2009), and it exhibited 50% QS inhibition activity against *C. violaceum* at a concentration of 295 nM. However, O'Loughlin et al. (2013) proved that it had a weak QS inhibition effect in *P. aeruginosa* (Swem et al., 2009; O'Loughlin et al., 2013). Compared with 1,3-benzoxazol-2(3 H)-one derivatives, compound no.10 was able to achieve the similar QS-inhibition activity at lower concentrations (Miandji et al., 2012).

Based on aforementioned results, we supposed that the phenotype of QS system inhibition was attributed to the interaction between compounds and LasR. Molecular docking results have shown that compound no.1 interacted with LasR by formation of hydrogen bonds with three sites: Try 56, Trp 60, and Asp 73. While both the compound no.3 and compound no.10 interacted with receptors at four sites: Try 56, Trp 60, Asp 73, and Ser 129, consistent with the binding site of the natural ligand (Abbas et al., 2017). A previous study has shown that the absence of the hydrogen bonds donated by Ser 129 in the LasR ligand-binding pocket may result in the reduction of QS inhibition activity of ligands (Manson et al., 2020). It was proved that Ser 129 of LasR may be an essential site for QS inhibitor binding to LasR. Moreover, the Lavina scores of compound no.3 and no.10 are −9 and −7, respectively, which indicated a high affinity of compound no.10 to bind with LasR, and it could contribute to its anti-QS activity.

In a previous study, biofilm formation related to the LasI/R system and RhlI/R system was confirmed, which is responsible for biofilm differentiation and optimal biofilm formation, respectively (Favre-Bonté et al., 2003). While virulence factors, such as pyocyanin and elastase, also were controlled by PQS and RhlI/R system (Oliveira et al., 2016; McCready et al., 2019). To further explore the compounds’ mechanism, the relative expressions of QS-regulated (*lasI*, *lasR*, *rhlI*, *rhlR*, and *mvfR*) genes were selected to be tested (Kim et al., 2015). It has shown that compound no.3 and compound no.10 effectively downregulate *lasI*, *lasR*, rhlI, *rhlR*, and *mvfR*, while compound no.1 slightly inhibited the expression of *lasI*, *rhlI*, and *rhlR*. These results illustrated the possible causes that compound no.3 and compound no.10 had more significant bioactivity of anti-biofilm and anti-virulence at the gene level. In addition, compound no.10 has shown the more significant effect on downregulating the expression of QS-regulated genes than compound no.3, especially reduction in the expression of *mvfR*, which is the most important regulator of virulence production (Cao et al., 2001).

Based on aforementioned results, the structure–activity relationship trends influencing inhibitory activity are provided as follows: 1) AHL analog aromatic functionality with electron-withdrawing groups had stronger QS inhibition activity. 2) AHL analogs with electron-withdrawing and lipophilic substituents in the 4th position on the phenyl group displayed the stronger antagonistic activities than other analogs. 3) AHL analogs with an elastic spacer flexible carbon spacer of at least one carbon between the homoserine lactone ring and the benzene ring showed optimal inhibition activity.

In addition, our work proved that the substitutes of natural acyl group can be used as the strategy for synthesizing QS inhibitor. Moreover, our compounds had no stress on neither bacterial nor normal mammalian cell growth (Supplementary Figure S1); it is safe and can avoid the development of drug resistance.

In conclusion, 2-(4-bromophenyl)-N-(2-oxotetrapyridinefuran-3-yl) butanamide (compound no.10) could be considered as the most promising anti-QS compound against *P. aeruginosa*. Nevertheless, further experiments are conducted to evaluate the effect of compound no.10 on clinical drug-resistant strains and assess the therapeutic effect of compound no.10 on the infected animal model. In addition, further experiments are conducted to explore the QS inhibition mechanism of compound no.10.

## Data Availability

The original contributions presented in the study are included in the article/Supplementary Material; further inquiries can be directed to the corresponding author.
