# Supplementary material for: Design, Synthesis, and Biological Evaluation of N-Acyl-Homoserine Lactone Analogs of Quorum Sensing in Pseudomonas aeruginosa
Source: Front Chem. 2022 Jul 8;10:948687. doi: 10.3389/fchem.2022.948687 (PMC9305322; doi:10.3389/fchem.2022.948687)
Supplement: Supplementary file 1 [file DataSheet1.docx]

Supplementary Material

**Table 1 1 H NMR and 13 C NMR data of AHL and analogues**

| Compound | 1 H NMR (DMSO-d6, 400 MHz) δ | 13C NMR (DMSO-d6, 100 MHz) δ |
| --- | --- | --- |
| NO.1 | 8.62 (d, 1H, NH, J=7.6 Hz), 7.50 (d,2H, Ar-H, J=8.0 Hz), 7.22 (d, 2H, Ar-H, J=8.4 Hz), 4.59-4.52 (m, 1H, CHNH), 4.33 (m, 1H, CHH'OC(=O)), 4.23-4.16 (m, 1H, CHH'OC(=O)), 3.45 (s, 2H, C(=O) CH2), 2.42-2.35 (m, 1H, CHH'CHNH), 2.15-2.09 (m, 1H, CHH'CHNH). | 175.3 (C=O, ester), 169.8 (C=O, amide), 135.1, 131.3 (2C), 131.1 (2C), 119.7, 65.3, 48.0, 41.1, 28.2; Anal. calcd For C12H12NO3Br: C,48.34; H, 4.06; N, 4.70. |
| NO.2 | 8.62 (d, 1H, NH, J=8.0 Hz), 7.37 (d, 2H, Ar-H, J=8.4 Hz), 7.28 (d, 2H, Ar-H, J=8.4 Hz), 4.56 (m, 1H, CHNH), 4.35-4.31 (m, 1H, CHH'OC(=O)), 4.23-4.18 (m, 1H, CHH'OC(=O)), 3.47 (s, 2H, C(=O) CH2), 2.42-2.35 (m, 1H, CHH'CHNH), 2.15-2.10 (m, 1H, CHH'CHNH). | 175.2 (C=O, ester), 169.8 (C=O, amide), 134.8, 131.2 (2C), 130.9 (2C), 128.1, 65.2, 48.0, 41.0, 28.2. Anal. calcd For C12H12NO3Cl: C,56.81; H, 4.77; N, 5.52. Found: C, 56.79; H, 4.80; N, 5.50. |
| NO.3· | 9.20 (d, 1H, NH, J=7.6 Hz), 8.66 (d, 2H, Ar-H, J=8.8 Hz), 8.01 (d, 2H, Ar-H, J=8.4 Hz), 5.05 (m, 1H, CHNH), 4.83-4.78 (m, 1H, CHH'OC(=O)), 4.70-4.64 (m, 1H, CHH'OC(=O)), 3.81 (s, 2H, C(=O) CH2), 2.90-2.84 (m, 1H, CHH'CHNH), 2.63-2.58 (m, 1H, CHH'CHNH). | 175.1 (C=O, ester), 169.1 (C=O, amide), 146.3, 143.9, 130.7 (2C), 123.3 (2C), 65.3, 48.1, 41.4, 28.2. Anal. Calcd For C12H12N2O5: C, 54.55; H, 4.58; N, 10.60. |
| NO.4 | 8.69 (d, 1H, NH, J=7.6 Hz), 7.68 (d, 2H, Ar-H, J=8.0 Hz), 7.48 (d, 2H, Ar-H, J=8.0 Hz), 4.57 (m, 1H, CHNH), 4.34-4.32 (m, 1H, CHH'OC(=O)), 4.23-4.17 (m, 1H, CHH'OC(=O)), 3.59 (s, 2H, C(=O) CH2), 2.43-2.39 (m, 1H, CHH'CHNH), 2.19-2.11 (m, 1H, CHH'CHNH). | 175.2 (C=O, ester), 169.5 (C=O, amide), 140.7, 129.9 (2C), 125.0 (2C), 65.3, 48.1, 41.5, 28.2. Anal. calcd For C13H12NO3F3: C, 54.36; H, 4.21; N, 4.88. Found: C, 54.40; H, 4.18; N, 4.90. |
| NO.5 | 8.55 (d, 1H, NH, J=8.0 Hz),7.15-7.09 (m, 4H, Ar-H), 4.54 (m, 1H, CHNH), 4.35-4.31 (m, 1H, CHH'OC(=O)), 4.22-4.16 (m, 1H, CHH'OC(=O)), 3.40 (s, 2H, C(=O) CH2), 2.41-2.34 (m, 1H, CHH'CHNH), 2.26 (s, 3H, CH3), 2.14-2.09 (m, 1H, CHH'CHNH). | 175.3 (C=O, ester), 170.3 (C=O, amide), 135.4, 132.7, 128.8 (4C), 65.2, 48.0, 41.5, 28.2, 20.6. Anal. calcd For C13H15NO3: C, 66.94; H, 6.48; N, 6.00. Found: C, 67.00; H, 6.45; N, 5.95. |
| NO.6 | 8.53 (d, 1H, NH, J=8.0 Hz), 7.17 (d, 2H, Ar-H, J=8.4 Hz), 6.86 (d, 2H, Ar-H, J=8.4 Hz), 4.54 (m, 1H, CHNH), 4.35-4.31 (m, 1H, CHH'OC(=O)), 4.22-4.18 (m, 1H, CHH'OC(=O)), 3.72 (s, 3H, OCH3), 3.38 (s, 2H, C(=O) CH2), 2.41-2.34 (m, 1H, CHH'CHNH), 2.14-2.09 (m, 1H, CHH'CHNH). | 175.3 (C=O, ester), 170.5 (C=O, amide), 157.9, 130.0 (2C), 127.7, 113.7 (2C), 65.2, 55.0, 48.0, 41.0, 28.2. Anal. calcd For C13H15NO4: C, 62.64; H, 6.07; N, 5.62. Found: C, 62.60; H, 6.10; N, 5.63. |
| NO.7 | 8.61 (d, 1H, NH, J=7.6 Hz), 7.59 (d, 1H, Ar-H, J=7.6 Hz), 7.39-7.32 (m, 2H, Ar-H), 7.22-7.18 (m, 1H, Ar-H), 4.58-4.53 (m, 1H, CHNH), 4.36-4.32 (m, 1H, CHH'OC(=O)), 4.24-4.19 (m, 1H, CHH'OC(=O)), 3.63 (s, 2H, C(=O)CH2), 2.42-2.39 (m, 1H, CHH'CHNH), 2.19-2.14 (m, 1H, CHH'CHNH). | 175.1 (C=O, ester), 168.9 (C=O, amine), 135.5, 132.2, 131.8, 128.7, 127.5, 124.4, 65.2, 48.0, 42.0, 28.1. Anal. calcd For C12H12NO3Br: C, 48.34; H, 4.06; N, 4.70. Found: C, 48.51; H, 4.03; N, 4.70. |
| NO.8 | 8.64 (d, 1H, NH, J=7.6 Hz), 7.48-7.43 (m, 2H, Ar-H), 7.30-7.26 (m, 2H, Ar-H), 4.59-4.52 (m, 1H, CHNH), 4.36-4.32 (m, 1H, CHH'OC(=O)), 4.23-4.17 (m, 1H, CHH'OC(=O)), 3.49 (s, 2H, C(=O)CH2), 2.43-2.36 (m, 1H, CHH'CHNH), 2.16-2.11 (m, 1H, CHH'CHNH). | 175.2 (C=O, ester), 169.7 (C=O, amide), 138.5, 131.8, 130.4, 129.4, 128.2, 121.4, 65.3, 48.1, 41.2, 28.2. Anal. calcd For C12H12NO3Br: C,48.34; H, 4.06; N, 4.70. Found: C, 48.45; H, 4.02; N, 4.73. |
| NO.9 | 8.40 (d, 1H, NH, J=8.0 Hz), 7.45 (d, 2H, Ar-H, J=8.0 Hz), 7.18 (d, 2H, Ar-H, J=8.0 Hz), 4.53 (m, 1H, CHNH), 4.34 (m, 1H, CHH'OC(=O)), 4.22-4.19 (m, 1H, CHH'OC(=O)), 2.79 (t, 2H, CH2CH2C(=O), J=8.0 Hz), 2.42-2.32 (m, 3H, CHH'CHNH, CH2CH2C(=O)), 2.11-2.06 (m, 1H, CHH'CHNH). | 175.3 (C=O, ester), 171.2 (C=O, amide), 140.5 (C-1), 131.0 (2C, C-3, C-5), 130.6 (2C, C-2, C-6), 118.9 (C-4), 65.2 (C-5'), 47.8 (C-3'), 36.3 (CH2), 30.1 (CH2), 28.1 (C-4'). Anal. calcd For C13H14NO3Br: C, 50.02; H, 4.52; N, 4.49. Found: C, 50.05; H, 4.55; N, 4.50. |
| NO.10 | 8.34 (d, 1H, NH, J=7.6 Hz), 7.46 (d, 2H, Ar-H, J=8.0 Hz), 7.16 (d, 2H, Ar-H, J=8.0 Hz), 4.56-4.49(m, 1H, NHCH), 4.36-4.31 (m, 1H, CHH′OC(=O)), 4.13-4.26 (m, 1H, CHH′OC(=O)), 2.56-2.50 (m, 2H, CH2CH2CH2C(=O)NH), 2.40-2.34 (m, 1H, CHH′CHNH), 2.16-2.09 (m, 3H, CHH′CHNH, CH2CH2CH2C(=O)NH), 1.81-1.74 (m, 2H, CH2CH2CH2C(=O)NH). | 175.6 (C=O, ester), 172.1 (C=O, amide), 141.2, 131.2 (2C), 130.8 (2C), 118.9, 65.4, 48.0, 34.3, 33.7, 28.2, 26.8; Anal. calcd For C14H16NO3Br: C, 51.55; H. 4.94; N, 4.29. Found: C, 51.60; H, 4.90; N, 4.30. |

**Table 2 Primers used in this study**

| Primer name | Gene | Oligonucleotide sequences (5′→3′) | Tm (°C) | GC% |
| --- | --- | --- | --- | --- |
| lasR-F | lasR | ACGCTCAAGTGGAAAATTGG | 60.11 | 45.00 |
| lasR-R | lasR | GTAGATGGACGGTTCCCAGA | 59.93 | 55.00 |
| lasI-F | lasI | CTACAGCCTGCAGAACGACA | 60.20 | 55.00 |
| lasI-R | lasI | ATCTGGGTCTTGGCATTGAG | 60.07 | 50.00 |
| rhlR-F | rhlR | AGGAATGACGGAGGCTTTTT | 60.07 | 45.00 |
| rhlR-R | rhlR | CCCGTAGTTCTGCATCTGGT | 60.13 | 55.00 |
| rhlI- F | rhlI | CTCTCTGAATCGCTGGAAGG | 60.09 | 55.00 |
| rhlI- R | rhlI | GACGTCCTTGAGCAGGTAGG | 59.87 | 60.00 |
| mvfR-F | mvfR | AACCTGGAAATCGACCTGTG | 59.97 | 50.00 |
| mvfR-R | mvfR | TGAAATCGTCGAGCAGTACG | 60.01 | 50.00 |
| 16srRNA-F | 16srRNA | GCGCAACCCTTGTCCTTAGTT | 57.80 | 52.38 |
| 16srRNA-R | 16srRNA | TGTCACCGGCAGTCTCCTTAG | 59.70 | 57.14 |


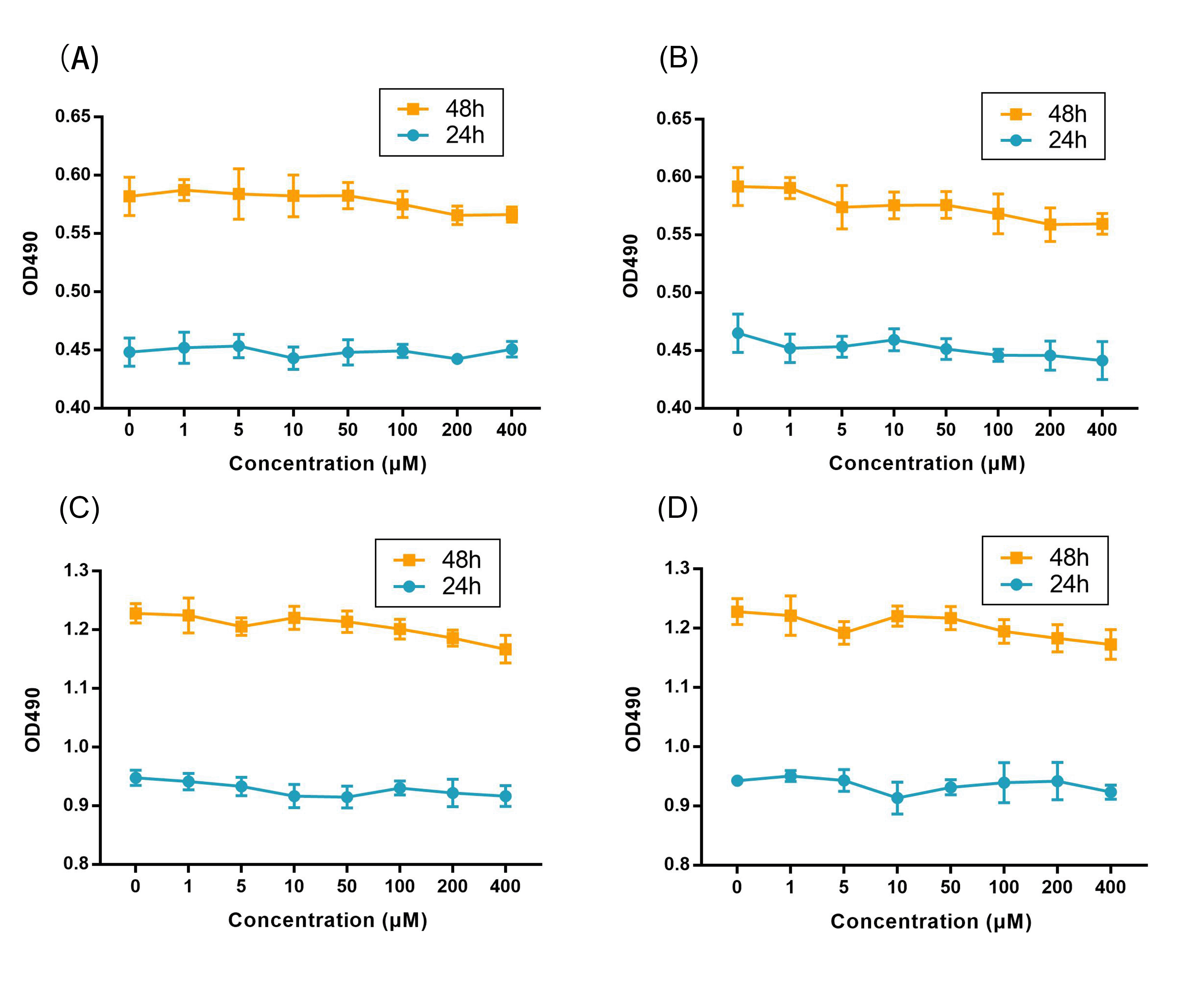


Figure S1. Effect of different concentrates of Compounds on the viability of BEAS-2B and HL7702. (A) and (C) were BEAS-2B with exposure of Compound NO.4 and Compound NO.13, respectively. (B) and (D) were HL7702 with exposure of Compound NO.4 and Compound NO.13, respectively. All data are representative of three independent experiments performed in triplicate and expressed as the mean ± SD values in each bar.
